# Duane’s Students’ Dictionary of Medicine

**Published:** 1894-02

**Authors:** 


### Book Reviews.

---

Duane's Students' Dictionary of Medicine. The Students' Dictionary of Medicine and the Allied Sciences. Comprising the Pronunciation, Derivation and Full Explanation ot Medical Terms, together with much collateral descriptive matter, numerous tables, etc. By Alexander Duane, M. D., Assistant Surgeon to the New York Ophthalmic and Aural Institute; Reviser of Medical Terms for Webster's International Dictionary. In one square octavo volume of 658 pages. Cloth, $4 25; half leather, $4.50; full sheep, $5.00. Philadelphia, Lea Brothers & Co., 1893.

A work designed for the medical student; made as compact as possible by the omission of obsolete and rare and unused words. Pronunciation is indicated by a phonetic spelling rather than by diacritical marks. The author has not confined himself to simple definitions, but has introduced much explanatory and descriptive matter.

Under each drug are given its action and therapeutic uses together with its official preparations. Definitions of the principal diseases are accompanied by a sketch of their etiology, symptoms and treatment. Much matter not found in the ordinary text-books is given in tabular form; the arteries, canals, nerves and muscles are thus arranged. There are also excellent tables of the bacteria, of fermentations, etc. These tables have been compiled with care and should afford material assistance to the student and constitute a new and valuable addition to a student's dictionary.

